# Argon plasma coagulation under direct peroral cholangioscopy for intraductal papillary mucinous neoplasm of the gallbladder

**DOI:** 10.1055/a-2094-9526

**Published:** 2023-06-22

**Authors:** Kyong Joo Lee, Se Woo Park, Eun Shin, Dong Hee Koh, Jin Lee

**Affiliations:** 1 Division of Gastroenterology, Department of Internal Medicine, Hallym University Dongtan Sacred Heart Hospital, Hallym University College of Medicine, Hwaseong, South Korea; 2 Department of Pathology, Hallym University Dongtan Sacred Heart Hospital, Hallym University College of Medicine, Hwaseong, South Korea

A gallbladder intraductal papillary neoplasm, similar to a bile duct and pancreas intraductal papillary mucinous neoplasm (IPMN), is a rare premalignant lesion characterized by superﬁcial spread, dilated gallbladder and bile ducts, and multifocal distribution 1 2 .

A 91-year-old woman presented to the emergency department with jaundice. An abdominal computed tomography scan revealed a markedly dilated cystic duct and intra- and extrahepatic bile ducts, without visible stones or masses ( Fig. 1 a ). On endoscopic retrograde cholangiopancreatography (ERCP), abundant mucin exuding from papilla appeared as fisheye signs, and amorphous ﬁlling defects occupying the extrahepatic duct, consistent with mucobilia ( Fig. 1b ), were observed. The patient refused surgery because of her current condition and extremely old age. Despite multiple sessions of ERCP with mucobiliary clearance, she experienced recurrent episodes of obstructive cholangitis resulting from mucus impaction. After initial failure of direct peroral cholangioscopy using an ultrathin endoscope for clearance, a standard upper endoscope was introduced into the extrahepatic duct and inserted into the hilum ( Video 1 ). After sufficient mucus suction, direct peroral cholangioscopy demonstrated multiple exophytic papillary protrusions in the cystic duct and gallbladder ( Fig. 2 a ). Histological evaluation of the specimens revealed IPMN with low-grade dysplasia in the gallbladder nodularities ( Fig. 2 b ). To prevent recurrent cholangitis by reducing the tumor burden and mucin production, argon plasma coagulation (APC) was performed. After two sessions of direct peroral cholangioscopy, APC was applied to all visible nodularities in the gallbladder and cystic duct with a pulsed mode of 40 watts, effect 1, and 1.0 L/min. Intermittent suction was followed by APC to reduce gas overdistention. At follow-up, direct peroral cholangioscopy showed a signiﬁcant reduction of mucobilia and fibrotic scar changes. The remaining nodules were treated with APC in the third direct peroral cholangioscopy session. Cholangitis was not observed after endoscopic therapy.

**Fig. 1:**
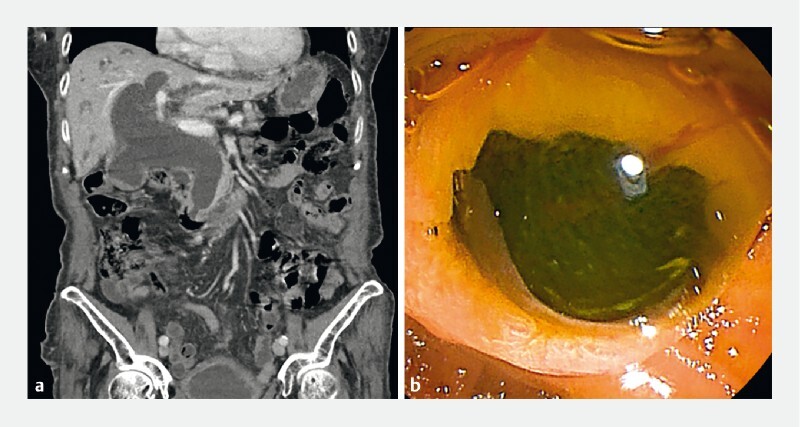
Initial abdominal computed tomography (CT) and endoscopic findings. **a** Initial abdominal CT scan reveals a markedly dilated cystic duct and intra- and extrahepatic bile ducts. **b** Endoscopic image showing abundant mucin and fisheye signs.

**Video 1** Argon-plasma coagulation was performed at papillary lesions to reduce tumor burden for reducing mucin production during 2nd session of direct peroral cholangioscopy. The lesions were successfully ablated.


**Fig. 2:**
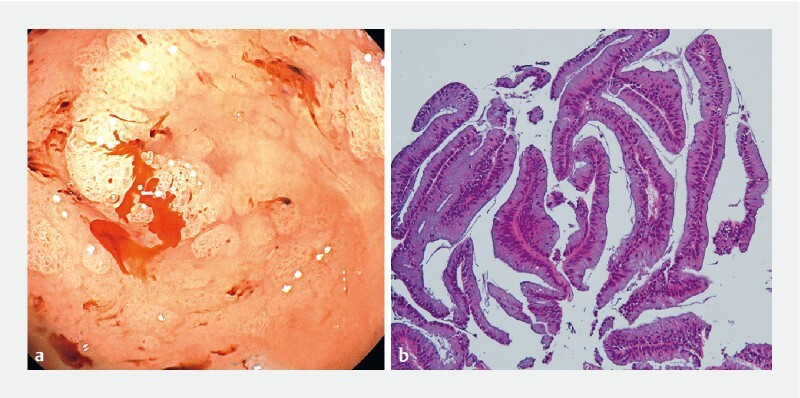
Endoscopic and pathologic findings of the gallbladder. **a** Endoscopic image showing multiple exophytic papillary protrusions. **b** Hematoxylin and eosin staining (× 100) showing an intraductal papillary mucinous neoplasm with low-grade dysplasia.

Although surgery is the treatment of choice for gallbladder IPMN, surgically unfit patients beneﬁt from minimally invasive endoscopic therapies, including APC 3 4 .

Endoscopy_UCTN_Code_TTT_1AR_2AF

## Notes

Endoscopy E-Videos

https://eref.thieme.de/e-videos
*E-Videos* is an open access online section of the journal *Endoscopy* , reporting on interesting cases and new techniques in gastroenterological endoscopy. All papers include a high-quality video and are published with a Creative Commons CC-BY license. Endoscopy E-Videos qualify for HINARI discounts and waivers and eligibility is automatically checked during the submission process. We grant 100% waivers to articles whose corresponding authors are based in Group A countries and 50% waivers to those who are based in Group B countries as classified by Research4Life (see: https://www.research4life.org/access/eligibility/ ). This section has its own submission website at https://mc.manuscriptcentral.com/e-videos

## References

[1] Adsay V Jang K T Roa J C Intracholecystic papillary-tubular neoplasms (ICPN) of the gallbladder (neoplastic polyps, adenomas, and papillary neoplasms that are >/=1.0 cm): clinicopathologic and immunohistochemical analysis of 123 cases Am J Surg Pathol 2012 36 1279 1301 22895264 10.1097/PAS.0b013e318262787c

[2] Zen Y Fujii T Itatsu K Biliary papillary tumors share pathological features with intraductal papillary mucinous neoplasm of the pancreas Hepatology 2006 44 1333 1343 17058219 10.1002/hep.21387

[3] Syed A R Kumar U Garg M Argon plasma coagulation treatment of intraductal papillary neoplasm of biliary tract: an alternative approach VideoGIE 2018 3 234 235 30128400 10.1016/j.vgie.2018.03.006 PMC6095452

[4] Cha B Park J S Jeong S Direct cholangioscopy with argon plasma coagulation of an intraductal papillary mucinous neoplasm of the bile duct Korean J Intern Med 2019 34 940 941 29761795 10.3904/kjim.2017.301 PMC6610195

